# Associations between autonomic nervous system activity and risk‐taking and internalizing behavior in young adolescents

**DOI:** 10.1111/psyp.13882

**Published:** 2021-06-18

**Authors:** Lisa Loheide‐Niesmann, Tanja G. M. Vrijkotte, Susanne R. De Rooij, Reinout W. Wiers, Anja Huizink

**Affiliations:** ^1^ Department of Public and Occupational Health Amsterdam UMC, Location AMC, University of Amsterdam Amsterdam The Netherlands; ^2^ Department of Clinical, Neuro‐ & Developmental Psychology Vrije Universiteit Amsterdam Amsterdam The Netherlands; ^3^ Behavioural Science Institute Radboud University Nijmegen The Netherlands; ^4^ Department of Epidemiology and Data Science Amsterdam UMC, Location AMC, University of Amsterdam Amsterdam The Netherlands; ^5^ Addiction Development and Psychopathology (ADAPT)‐Lab Department of Psychology University of Amsterdam Amsterdam The Netherlands

**Keywords:** adolescents, heart rate, internalizing, parenting, risk‐taking, RSA

## Abstract

Dysregulated autonomic nervous system (ANS) activity has been associated with adolescent risk‐taking and internalizing behavior, but previous results in community samples have been mixed. We investigated whether ANS activity was associated with higher risk‐taking and internalizing behavior in young adolescents (age 11/12; *n* = 875), and whether adolescents' gender, parents' parenting style or a combination of both moderated these associations. Adolescents and their parents were recruited as part of the population‐based, longitudinal Amsterdam Born Children and their Development (ABCD) study. Risk‐taking behavior was assessed with the Balloon Analogue Risk Task and the personality characteristics sensation seeking and impulsivity, measured with the Substance Use Risk Profile Scale (SURPS). Internalizing behavior was assessed via the SURPS subscales anxiety sensitivity and hopelessness. Authoritative (AUTH‐SW) and authoritarian (AUTH‐S) parenting styles were measured with the Parenting Styles and Dimensions Questionnaire. Resting ANS activity was assessed via heart rate and respiratory sinus arrhythmia (RSA). Hierarchical, multivariable regression analyses showed higher RSA, but not heart rate, being associated with higher risk‐taking behavior and sensation seeking. The associations between ANS activity and risk‐taking variables were not significantly moderated by gender, parenting, or interactions between gender and parenting. Our findings suggest that RSA activity may be a relevant factor in mild to moderate risk‐taking behavior in adolescents from the general population, regardless of their gender or the type of parenting they experience.

## Abstract 2

Dysregulated autonomic nervous system activity has been associated with adolescent risk‐taking and internalizing behavior, but previous results in community samples have been mixed. Our findings suggest that in adolescents from the general population, respiratory sinus arrhythmia activity may be a relevant factor in mild to moderate risk‐taking behavior but not in internalizing behavior, regardless of adolescents' gender or the type of parenting they experience.

## 1 INTRODUCTION

Adolescence is a challenging developmental period marked by behavioral, cognitive, and hormonal changes (Steinberg, 2005), requiring effective stress and emotion regulation (Turpyn et al., 2015). Adolescents who struggle with effectively regulating their emotions and coping with the stressors of adolescence are at risk for internalizing problems and are more likely to engage in risk‐taking behaviors like risky substance use or sexual behavior (Danneel et al., 2019; Silk et al., 2003; Stankovic et al., 2014).

Previous research has linked abnormal autonomic nervous system (ANS) activity to increased risk‐taking and internalizing problems (Beauchaine & Thayer, 2015; Evans et al., 2016; Goeders, 2003). The ANS supports individuals in responding to environmental threats or challenges. As such, the ANS plays a major role in the physiological stress response and in self‐ and emotion regulation (Beauchaine, 2001; Porges, 2007). Two major branches make up the ANS: the sympathetic nervous system, responsible for mobilizing the body in the face of challenges or threats; and the parasympathetic nervous system, which is mostly associated with calm states, restoration following stress responses and general homeostasis (Dietrich et al., 2007; Porges, 1995). One index of ANS activity is resting heart rate, reflecting the balance between sympathetic and parasympathetic activity (Burgess et al., 2003; Deutz et al., 2019; Dietrich et al., 2007). Resting respiratory sinus arrhythmia (RSA) indicates primarily parasympathetic activity and reflects variations in heart rate inter‐beat intervals across respiration (Porges, 2007). RSA has been interpreted as an index of emotion‐ and self‐regulation capacity, the degree to which individuals can react flexibly to environmental changes (Beauchaine, 2001; Porges, 1995).

Risk‐taking behaviors, such as aggressive or impulsive externalizing behaviors, have often been associated with lower resting or baseline heart rate (e.g., De Pascalis et al., 2007; Dietrich et al., 2007), while internalizing problems have been associated with higher heart rate (e.g., Behnsen et al., 2020; Byrne et al., 2010; Dietrich et al., 2007; Hastings et al., 2011). These findings might indicate that adolescents exhibiting higher risk‐taking may be hypo‐aroused, i.e., they may exhibit diminished physiological responses to stressors (Deutz et al., 2019; Hastings et al., 2011), and may therefore stimulate their arousal using risk‐taking behaviors. In contrast, internalizing problems in adolescents may indicate physiological overarousal (Dietrich et al., 2007; Monk et al., 2001; Woltering & Shi, 2016). However, previous research has been inconsistent, with studies failing to find different resting heart rate patterns for internalizing and risk‐taking or externalizing behavior, or finding no associations between heart rate and internalizing or externalizing behavior (Bimmel et al., 2008; Deutz et al., 2019; Dorn et al., 2003; Herman et al., 2019).

Resting or baseline RSA has been found to be lower in adolescents exhibiting internalizing or externalizing behavior, suggesting that both problem behaviors may be characterized by similarly dysregulated autonomic profiles, which reflect difficulties with self‐ and emotion regulation (e.g., Beauchaine, 2001; Beauchaine et al., 2019; Shader et al., 2018; Xu et al., 2014). However, research has mainly focused on male, clinical populations, particularly regarding externalizing behavior (Van der Graaff et al., 2016). Contradicting evidence has been found as well—mainly in community samples—relating externalizing behaviors to high RSA (Dietrich et al., 2007; Scarpa & Ollendick, 2003) or finding no associations between RSA and internalizing or externalizing behaviors (McLaughlin et al., 2015; Paysnick & Burt, 2015; Van der Graaff et al., 2016). Finally, few studies have investigated the link between ANS activity and general (non‐specific) risk‐taking, rather than (aggressive or impulsive) externalizing behavior. General risk‐taking, a maladaptive pattern of choice behavior in conditions with both reward and negative outcome potential, is related to various negative outcomes (Bennett et al., 2014; Lejuez et al., 2007). Since ANS activity might be differentially related to different types of externalizing behavior (Dietrich et al., 2007), it remains unclear whether previous findings on aggressive and antisocial risk‐taking behavior can be replicated for general risk‐taking.

The previous contradicting findings regarding ANS activity and risk‐taking and internalizing behaviors could be due to gender moderating associations between ANS activity and risk‐taking and internalizing behavior. Previous research has reported gender‐differential associations between ANS activity and externalizing and internalizing behavior in adolescents, but with conflicting results: associations between RSA and externalizing as well as internalizing problems were found both for boys only (Aults et al., 2015; Beauchaine et al., 2008; El‐Sheikh & Hinnant, 2011) and for girls only (Dietrich et al., 2007; Tu et al., 2019). Regarding heart rate, Allen et al. (2009) reported associations between heart rate and impulsivity in boys only, albeit in older adolescent and young adult university students. Gender differences have also been found regarding general ANS activity (McLaughlin et al., 2015) and risk‐taking and internalizing problems, with the latter findings indicating that the development of behavior problems of girls and boys could involve different mechanisms (Beauchaine et al., 2008; Zhang et al., 2017).

Additionally, environmental influences (Khurshid et al., 2019) and particularly parental influences might affect associations between ANS activity and risk‐taking and internalizing behavior (Stanger et al., 2016). Given that parenting behavior can impact adolescents' risk‐taking and internalizing symptoms but also their ANS activity (Bell et al., 2018; DeVore & Ginsburg, 2005; Evans et al., 2013; Pinquart, 2017a, 2017b; Sangawi et al., 2018), it could moderate associations between (inherently) dysregulated ANS activity and risk‐taking or internalizing behavior. Authoritative parenting (AUTH‐SW), characterized by strictness and warmth (SW), could protect against risk‐taking and internalizing symptoms (Calafat et al., 2014; Williams et al., 2009), possibly because authoritative parents may teach their (dysregulated) adolescents adaptive coping strategies for their vulnerabilities. Authoritarian parenting (AUTH‐S) (strictness and no warmth), however, was associated with higher internalizing and risk‐taking symptoms (Williams et al., 2009), possibly because authoritarian parents may increase adolescents' stress without teaching them healthy coping strategies. In line with this, previous research found externalizing behavior, reactive aggression, and delinquency to be highest in boys with lower baseline RSA who also experienced negative parenting, i.e., harsh or inconsistent parenting, or low levels of authoritative parenting (Dyer et al., 2016; Hinnant et al., 2015; Kassing et al., 2018). Also, Cai and Tu (2020) reported that in adolescents of mothers using high levels of psychological control, a form of negative parenting, lower baseline RSA was associated with higher internalizing symptoms in boys and girls, whereas boys with higher baseline RSA did not exhibit increased internalizing symptoms. To date, however, few studies have examined interactions between resting RSA and parenting styles on adolescent problem behavior (Cai & Tu, 2020).

The proposed moderating effects of parenting may also differ by parent and adolescent gender. According to previous research, mothers use more authoritative but less authoritarian parenting than fathers (Conrade & Ho, 2007). Moreover, mothers and fathers parent sons and daughters differently: mothers reason more with their daughters but show some favoritism for their sons while sons perceived fathers as more authoritarian than daughters (Conrade & Ho, 2007). Additionally, maternal parenting influences daughters more strongly, whereas paternal parenting influences particularly boys (Barton & Kirtley, 2012). Gender differences in associations between parenting behavior and adolescent internalizing and externalizing behavior have also been found (Lansford et al., 2014). Finally, Cai and Tu (2020) found a three‐way interaction of gender, maternal psychological control and baseline RSA predicting adolescent loneliness and depressive symptoms over time. They found that boys with higher RSA, compared with boys with lower RSA, showed lower levels of loneliness and depression when maternal psychological control was low, and higher levels of loneliness and depression when psychological control was high. In girls, the associations between maternal psychological control and loneliness and depression were not affected by baseline RSA. These findings suggest that an interaction of adolescent gender and parenting styles could also moderate associations between ANS activity and internalizing and risk‐taking behavior.

The present study examined whether ANS activity (resting heart rate and RSA) was related to risk‐taking and internalizing behavior in young adolescents (age 11–12). Risk‐taking was measured via a behavioral task and the personality traits sensation seeking and impulsivity. Internalizing behavior was measured via the traits anxiety sensitivity and hopelessness, which are often associated with anxiety and/or depression (Horwitz et al., 2017; Rodriguez et al., 2004). In line with previous research, we hypothesized lower resting heart rate to be associated with higher risk‐taking behavior (hypothesis 1), whereas higher heart rate would be associated with higher internalizing behavior (hypothesis 2). Moreover, we expected lower RSA to be associated with higher risk‐taking (hypothesis 3) and also higher internalizing behavior (hypothesis 4). We also expected adolescent gender to moderate the associations between ANS activity and risk‐taking and internalizing behavior (hypothesis 5). Given conflicting previous findings, which have reported associations between ANS activity and risk‐taking and internalizing behavior both for boys only and for girls only (Beauchaine et al., 2008; Dietrich et al., 2007; El‐Sheikh & Hinnant, 2011; Tu et al., 2019), we did not formulate specific hypotheses about the direction of this expected moderating effect.

Additionally, we investigated moderating effects of parenting styles on associations between ANS activity and risk‐taking and internalizing behavior. We expected high AUTH‐SW parenting to have a protective effect, leading to low risk‐taking and internalizing behavior regardless of ANS activity (hypothesis 6). High AUTH‐S parenting was hypothesized to exacerbate particularly dysregulated adolescents' risk‐taking and internalizing behavior (hypothesis 7). Moreover, mothers' (hypothesis 8) and fathers' (hypothesis 9) parenting was expected to affect their respective same‐gender children more strongly.

## 2 METHOD

### 2.1 Study population and procedures

This study is part of the population‐based, longitudinal Amsterdam Born Children and their Development (ABCD) study, which received approval from the medical ethics review committees of the Academic Medical Center, Amsterdam, and the VU University Medical Center, Amsterdam. In the first ABCD study phase, which took place in 2003 and 2004, all pregnant women living in Amsterdam were invited to complete questionnaires, which 8,266 (66.8%) out of 12,373 women did. After giving birth, 6,375 women gave permission for follow‐up (for further details regarding the study procedure, see Van Eijsden et al., 2011).

The present study used data from the fourth ABCD phase, which was conducted in 2015 and 2016, when adolescents were 11–12 years old. Questionnaires were sent to 5,644 still participating families, which 2,997 (53.1%) mothers, 2,264 (40.1%) fathers, and 3,018 (53.5%) adolescents completed. Attrition between the study's first and fourth phase was largely due to migration or untraceable address changes. Parents gave written informed consent for themselves and their child, and 12‐year‐olds also provided consent themselves.

Questionnaires were mainly completed online, but paper questionnaires were also sent to families who had not completed any online questionnaires. The online questionnaires also included computer‐based behavioral tests. Additionally, a randomly selected subgroup of adolescents still participating in the ABCD study were invited for measurement days in central locations in Amsterdam, during which health measurements, including heart rate and RSA, were assessed by trained personnel. A total of 1,082 adolescents participated in these measurement days. For inclusion in the current study, data for at least one ANS and one outcome measure had to be available. Adolescents with heart problems or using medication affecting the ANS were excluded. Multiple births had already been excluded from the fourth ABCD phase. This resulted in a study sample of 875 adolescents (49.1% female, mean age = 11.8, *SD* = 0.37). Compared with adolescents who were excluded from this study due to incomplete data, the final study sample was significantly less pubertally mature (less hair growth in both sexes, less indications of growth spurt in girls) and consisted of a larger percentage of Dutch and a smaller percentage of non‐Western adolescents (all *p's* < .05).

### 2.2 Measures

#### 2.2.1 Autonomic nervous system activity

The VU University Ambulatory Monitoring System (VU‐AMS), whose use, reliability, and validity have been described elsewhere (Licht et al., 2013; Van Dijk et al., 2013), measured ANS activity in a standard procedure described elsewhere (Van Dijk et al., 2013), in which three‐lead electro cardiograms (ECG) and four‐lead impedance cardiograms were recorded. Assessment, during which adolescents were lying down, consisted of one minute of stabilization, followed by six minutes of measurement registration. Resting heart rate in beats per minute was obtained by extracting the interbeat interval time series from the ECG signal, which was manually checked for aberrances. Software automatically marked inspirations and expirations in the respiratory signal. Artifacts were labelled and any beats within periods labelled as artifacts were excluded from any further data analysis. RSA in milliseconds was obtained automatically by the VU‐AMS via peak‐valley estimation (Van Dijk et al., 2013).

#### 2.2.2 Risk‐taking behavior

Risk‐taking behavior was measured with the computerized Balloon Analogue Risk Task (BART; Lejuez et al., 2007), in which participants inflate balloons to earn points. After each pump, participants can transfer their points from a temporary bank to a permanent prize meter. If participants pump the balloon past its random explosion point, they lose all points in the temporary bank. All adolescents received a prize for their participation, but its size depended on the final number of points in the prize meter, to stimulate risk‐taking. Being a computerized task, the BART was only completed by adolescents completing questionnaires online. For analyses, the average number of pumps on unexploded balloons was used as a risk‐taking behavior index (Lejuez et al., 2007).

#### 2.2.3 Risk‐taking and internalizing behavior

The 23‐item Substance Use Risk Profile Scale (SURPS; Woicik et al., 2009) measured adolescent‐reported anxiety sensitivity, hopelessness, sensation seeking, and impulsivity. Anxiety sensitivity and hopelessness were used to assess internalizing behavior, whereas sensation seeking and impulsivity were used to assess risk‐taking behavior. Mean scores were calculated for each subscale. The SURPS has shown good concurrent, predictive, and discriminant validity (Krank et al., 2017; Woicik et al., 2009) and adequate to good internal reliability (*α* = .70–.90) (Woicik et al., 2009). In the present study, reliability was acceptable (*α* = .64–.78).

#### 2.2.4 Parenting styles

The 32‐item Parenting Styles and Dimensions Questionnaire‐32 (PSDQ‐32; Robinson et al., 1995) assessed parent‐reported authoritarian (AUTH‐S), authoritative (AUTH‐SW), and permissive parenting. The PSDQ‐32 has shown good concurrent and predictive validity and adequate to good reliability for the *authoritative* (*α* = .84–.86) and *authoritarian* subscales (*α* = .71–.75) in previous studies (Olivari et al., 2013; Robinson et al., 1995) and in the present study (AUTH‐SW: mothers = 0.85, fathers = 0.87; AUTH‐S: mothers = 0.75, fathers = 0.71). The *permissive* subscale was excluded from analyses due to its questionable validity (Kimble, 2014) and poor reliability (present study: mothers = 0.56; fathers = 0.52; previous studies: *α* = .38–.73; Kimble, 2014; Robinson et al., 1995). Sum scores were calculated for both subscales, separately for mothers and fathers.

#### 2.2.5 Demographic characteristics

Adolescents' age, gender, ethnicity, pubertal status (assessed with the Tanner scale; Tanner, 1962), parents' education level (four categories ranging from *a few years of primary school* to *college/university degree*), and financial situation (inadequate, adequate, more than adequate) were assessed via parent‐reported questionnaires.

### 2.3 Statistical analysis

Descriptive statistics were computed and correlations and simple associations between all study variables were analyzed. Associations between two continuous variables were analyzed with Pearson correlations, whereas associations involving one or two ordinal variables were analyzed using Spearman rank correlations. Gender differences in any of the independent or dependent study variables were analyzed using *t*‐tests. Any demographic variables that were associated with at least one ANS activity predictor or risk factor outcome were later added to the regression analyses as covariates. Demographic group differences between BART completers and non‐completers were analyzed using *t*‐tests and chi‐square tests. RSA was ln‐transformed using the natural logarithm, to normalize values and to obtain a better normal distribution approximation. Also, 2.1% of covariate data and 0.02% of RSA data were missing and were imputed using multiple imputation with the fully conditional specification method and 10 imputations.

Associations between ANS activity and risk factor outcomes were examined using hierarchical multivariable regression analyses. Individuals with missing outcome data were excluded from analyses including that outcome. Variables were centered for later moderation analyses and to reduce multicollinearity. In a first step, all covariates were added to the model, followed by heart rate or RSA, whose main effects were analyzed in separate models. Then, the moderating effects of gender and parenting styles were examined by adding interaction terms between adolescent gender and both ANS variables, and between the parenting and ANS variables into the regression analyses. Finally, three‐way interactions between adolescent gender, parenting variables, and ANS activity variables were computed and added to the regression analyses. Moderation analyses were adjusted for covariates and any significant interaction effects were further examined using simple slopes tests (Aiken et al., 1991). To reduce the number of false positive findings in our analyses, the false discovery rate (FDR) was controlled, separately for each study hypothesis (Rubin, 2017), by using the Benjamini‐Hochberg procedure, with the accepted false discovery rate set to 10% (McDonald, 2014). Both unadjusted and FDR‐adjusted results are reported.

## 3 RESULTS

### 3.1 General statistics

Descriptive statistics for covariates and for dependent and independent variables are presented in Tables 1 and 2. RSA could not be calculated by the VU‐AMS for 18 adolescents, because of more than 10% of the recording consisting of artifacts, due to irregularities in the respiration rate and interbeat intervals.

**TABLE 1:** Study population demographics, *n* = 875

	*n*	Frequency (%)	Mean (*SD*)
Gender (% girls)	875	49.3	
Age	873		11.8 (0.37)
Pubertal stage	863		
Girls: % menstruating	426	6.6	
Boys: % voice break	437	3.4	
Ethnicity	875		
% Dutch	667	76.2	
% Moroccan	23	2.6	
% Surinamese	34	3.9	
% Turkish	10	1.1	
% Other Non‐Western	43	4.9	
% Other Western	98	11.2	

**TABLE 2:** Descriptive statistics of all dependent and independent variables

Variables	Total study sample			Girls only			Boys only			*t*	*p*
	*n*	Mean	*SD*	*n*	Mean	*SD*	*n*	Mean	*SD*		
Heart rate (bpm)	875	72.05	9.14	431	73.82	9.05	444	70.34	8.91	−5.73	**<.001**
RSA (msec)	857	127.57	62.73	425	118.93	56.92	432	136.07	66.94	4.04	**<.001**
lnRSA	857	4.73	0.50	425	4.67	0.47	432	4.79	0.52	3.53	**<.001**
Risk‐taking	807	24.06	12.17	398	23.48	12.35	409	24.62	11.98	1.32	.19
Anxiety sensitivity	875	1.91	0.58	431	1.99	0.59	444	1.84	0.57	−3.81	**<.001**
Hopelessness	875	1.28	0.36	431	1.31	0.37	444	1.25	0.35	−2.40	.**02**
Sensation seeking	875	2.78	0.65	431	2.63	0.65	444	2.93	0.61	7.05	**<.001**
Impulsivity	875	1.89	0.63	431	1.82	0.60	444	1.95	0.64	2.92	.**004**
Authoritative parenting mothers	861	47.33	5.60	424	3.16	0.38	437	3.15	0.36	−0.59	.56
Authoritative parenting fathers	802	44.88	6.21	392	2.96	0.40	410	3.02	0.43	2.02	.**04**
Authoritarian parenting mothers	861	16.30	2.82	424	1.33	0.21	437	1.38	0.25	3.01	.**003**
Authoritarian parenting fathers	802	16.29	2.72	392	1.34	0.22	410	1.37	0.23	2.00	.**046**

Note

Bold *p*‐values denote statistical significance at the *p* < .05 level.

All adolescents completed the SURPS, but 68 adolescents did not complete the BART. BART non‐completers were older (*p* < .01) and financially poorer (*p* < .01) than completers, had lower educated parents (*p* < .01), were more pubertally mature (girls menstruating: *p* < .01; boys' voice break: *p* = .03), and fewer adolescents lived with both parents (*p* = .01). Non‐completers consisted of a lower percentage of Dutch and higher percentages of Surinamese, Moroccan, and other non‐Western adolescents (all *p's* < .01; see Table S5).

### 3.2 Correlational analysis

Heart rate and RSA correlated with each other (*r* = −.52, *p* < .01; Table 3) and with sensation seeking (HR: *r* = −.10, *p* < .01; RSA: *r* = .10, *p* < .01). Heart rate also correlated with anxiety sensitivity (*r* = .08; *p* < .05), and RSA correlated with risk‐taking (*r* = .09, *p* < .05). Neither ANS measure correlated with hopelessness or impulsivity. Except for ethnicity, all other covariates correlated with at least one ANS measure or outcome and were therefore controlled for in the subsequent regression analyses.

**TABLE 3:** Associations between all covariates, dependent, and independent variables

Variables	1	2	3	4	5	6	7	8	9	10	11	12	13	14	15	16	17	18
1. Age	–																	
2. Gender	0.04	–																
3. Ethnicity	0.02	0.02	–															
4. Pubertal stage	0.09*	0.79**	0.02	–														
5. Education mother^a^	−0.04^b^	−0.04^b^	−0.14^b^^,^ ^**^	−0.15^b^^,^ ^**^	–													
6. Education father^a^	−0.07^b^^,^ ^*^	0.00^b^	−0.12^b^^,^ ^**^	−0.11^b^^,^ ^**^	0.42^b^^,^ ^**^	–												
7. Financial situation^a^	−0.04^b^	0.02^b^	−0.14^b^^,^ ^**^	−0.06^b^	0.19^b^^,^ ^**^	0.25^b^^,^ ^**^	–											
8. Heart rate	−0.9**	0.19**	0.04	0.16**	−0.01^b^	−0.04^b^	−0.05^b^	–										
9. lnRSA^c^	0.01	−0.12**	0.02	−0.12**	0.03^b^	0.06^b^	0.04^b^	−0.52**	–									
10. BART	−0.03	−0.05	−0.02	−0.05	0.01^b^	0.08^b^^,^ ^*^	0.08^b^	−0.01	0.09*	–								
11. Anxiety Sensitivity	−0.2	0.13**	0.05	0.10**	−0.11^b^^,^ ^**^	−0.08^b^^,^ ^*^	−0.09^b^^,^ ^*^	0.08*	−0.05	−0.04	–							
12. Hopelessness	0.00	0.08*	0.00	0.06	−0.04^b^	−0.02^b^	−0.09^b^^,^ ^*^	0.03	−0.04	0.03	0.25**	–						
13. Sensation Seeking	−0.02	−0.23**	−0.05	−0.21**	0.05^b^	0.03^b^	0.03^b^	−0.10**	0.10**	0.10**	−0.11**	−0.04	–					
14. Impulsivity	0.01	−0.10**	−0.01	−0.07	−0.07^b^	−0.03^b^	−0.06^b^	0.00	0.01	0.03	0.29**	0.34**	0.17**	–				
15. AUTH‐SW mother	0.00	0.02	−0.03	0.06	0.04^b^	0.02^b^	0.06^b^	0.03	−0.01	0.04	0.00	−0.04	−0.01	0.12**	–			
16. AUTH‐SW father	−0.05	−0.07*	0.17**	−0.03	0.03^b^	0.05^b^	0.02^b^	−0.03	0.04	0.01	−0.02	−0.03	−0.04	0.28**	0.17**	–		
17. AUTH‐S mother	−0.04	−0.10**	0.03	−0.09**	−0.07^b^^,^ ^*^	−0.06^b^	−0.10^b^^,^ ^**^	0.03	0.03	0.01	0.09**	0.10**	0.10**	−0.08*	−0.15**	−0.12**	–	
18. AUTH‐S father	0.04	−0.07*	0.07	−0.07	−0.14^b^^,^ ^**^	−0.12^b^^,^ ^**^	−0.14**	0.06	0.02	0.01	−0.01	0.08*	0.07*	0.16**	−0.10**	−0.19**	0.38**	–

^a^ Pearson correlation coefficients were calculated, unless one or both of the variables to be correlated were ordinal variables, in which case Spearman rank correlations were used instead.

^b^ Spearman rank correlation

^c^ RSA was transformed using the natural logarithm (ln)

* Correlation is significant at the 0.05 level

** Correlation is significant at the 0.01 level

### 3.3 ANS activity and risk‐taking and internalizing behavior

Multiple regression analyses adjusted for demographic covariates revealed that heart rate was not associated with any risk factor outcome (Table 4). Higher RSA was associated with higher risk‐taking (*R* ^2^ = .02; *R* ^2^ change = .01; *F*(7, 799) = 2.15, *p* = .03) and sensation seeking (*R* ^2^ = .06; *R* ^2^ change = .01; *F*(7, 874) = 7.81, *p* < .001), and both associations remained significant when controlling the FDR using the Benjamini‐Hochberg procedure.

**TABLE 4:** Summary of multivariable hierarchical regression analyses, predicting young adolescents' risk‐taking and internalizing behavior with ANS activity. Heart rate and RSA were analyzed in separate regression models

ANS activity	Risk‐taking behavior						Internalizing behavior			
	Risk‐taking (BART)		Sensation seeking		Impulsivity		Anxiety sensitivity		Hopelessness	
	*B* (*SE*)	*p*	*B* (*SE*)	*p*	*B* (*SE*)	*p*	*B* (*SE*)	*p*	*B* (*SE*)	*p*
Heart rate	0.003 (0.048)	.94	−0.004 (0.002)	.08	0.001 (0.002)	.67	0.004 (0.002)	.10	0.000 (0.001)	.86
RSA	1.889 (0.863)	.**03** ^a^	0.090 (0.043)	.**04** ^a^	0.000 (0.044)	.99	−0.031 (0.039)	.43	−0.020 (0.025)	.42

Note

B = unstandardized coefficient; 95% CI = 95% confidence interval. Analyses are adjusted for age, gender, pubertal stage, parental education, and financial status. Bold *p*‐values denote statistical significance at the *p* < .05 level.

^a^ Both *p*‐values remain significant when controlling the false discovery rate using the Benjamini‐Hochberg procedure.

### 3.4 Moderation analyses

#### 3.4.1 Gender as a moderator in the associations between ANS activity and risk‐taking and internalizing behavior

None of the associations between ANS activity and risk‐taking or internalizing behavior were moderated by adolescent gender, when controlling the FDR (The results of the moderation analyses before controlling the FDR can be found in the supplemental materials).

#### 3.4.2 Parenting styles as moderating variables in the associations between ANS activity and risk‐taking and internalizing behavior

None of the associations between adolescent ANS activity and risk‐taking or internalizing behavior were significantly moderated by mothers' or fathers' AUTH‐SW or AUTH‐S parenting, when controlling the FDR.

#### 3.4.3 Interactions between adolescent gender and parenting styles as moderators in the associations between ANS activity and risk‐taking and internalizing behavior

None of the three‐way interactions including adolescent gender and mothers' or fathers' AUTH‐SW or AUTH‐S parenting were significant, when controlling the FDR. Neither mothers' nor fathers' parenting did thus have a significantly stronger effect on adolescents of the same gender.

## 4 DISCUSSION

In the present study, higher resting RSA was significantly associated with higher risk‐taking behavior (BART) and sensation seeking in young adolescents but not with impulsivity or with anxiety sensitivity or hopelessness. Heart rate was not associated with risk‐taking behavior or the risk‐taking behaviors impulsivity or sensation seeking, and heart rate was not associated with the internalizing behaviors anxiety sensitivity or hopelessness either. Neither gender or parenting styles nor a combination of gender and parenting styles significantly moderated any associations between ANS activity and risk‐taking or internalizing behavior.

The findings of higher risk‐taking and sensation seeking being associated with *higher* resting RSA contradicts previous theories and findings of externalizing, risk‐taking behaviors being associated with lower RSA in high‐risk or clinical populations (Beauchaine, 2001; Beauchaine et al., 2019; Shader et al., 2018), but are in line with findings of higher parasympathetic activity in relation to risk‐taking and externalizing behavior in community samples (Dietrich et al., 2007; Scarpa & Ollendick, 2003). The present findings also further highlight the differences between risk‐taking, externalizing behavior and their correlates in community samples compared to high‐risk, clinical samples (Dietrich et al., 2007). Notably, mild to moderate risk‐taking and externalizing behaviors are relatively normative in adolescents (Steinberg, 2004) and may thus be a distinct, non‐psychiatric construct as compared to severe externalizing behavior (Beauchaine & Thayer, 2015), which in turn may mean that these two constructs have different correlates and constructs. However, Dietrich et al. (2007) found higher resting RSA in adolescents exhibiting externalizing behavior who had exhibited similar behavior in preschool as well, indicating that their study sample's externalizing behavior included early‐starting externalizing problems that can thus not simply be classified as normative adolescent risk‐taking. While likely not as severe as in clinical populations, adolescents in community populations might still experience persistent risk‐taking and externalizing behavior problems and these behavior problems appear to be more likely to be associated with higher rather than lower resting RSA. Investigating the potential role of higher resting RSA as a marker for identifying adolescents at risk of high risk‐taking behavior in community settings might therefore be a promising avenue for future longitudinal research.

The lack of findings regarding associations between resting RSA and internalizing behavior and between resting heart rate and risk‐taking or internalizing behavior is in line with some previous findings in community samples (Bimmel et al., 2008; Dorn et al., 2003; McLaughlin et al., 2015; Paysnick & Burt, 2015), but contradicts other studies, with both clinical and community samples (Byrne et al., 2010; Dietrich et al., 2007). This contradictory literature emphasises the need for further research into the associations between heart rate and RSA on the one hand and risk‐taking and internalizing behavior. Particularly looking at mediators or moderators in the relationship between ANS activity and risk‐taking and internalizing behavior might promising for better understanding under which conditions and in which samples associations between heart rate or RSA and internalizing or risk‐taking behaviors can be found.

We therefore examined the moderating role of adolescent gender, mothers' and fathers' AUTH‐SW and AUTH‐S, and the combination of gender and parenting. Although we did find gender differences in heart rate, RSA, and all risk‐taking and internalizing variables except for risk‐taking behavior measured with the BART, gender did not emerge as a significant moderator when correcting for multiple testing. This contradicts previous findings of moderation effects of gender in associations between ANS activity and risk‐taking and internalizing behavior (Aults et al., 2015; Beauchaine et al., 2008; Dietrich et al., 2007; El‐Sheikh & Hinnant, 2011; Tu et al., 2019). Given that previous findings on gender‐differential associations between ANS activity and risk‐taking or internalizing behavior have been mixed, these effects might only exist in a subgroup of adolescents or only when assessing specific types of risk‐taking or internalizing behavior. Notably, our risk‐taking measures also differed from most previous studies; we assessed slightly different concepts, such as general risk‐taking rather than aggressive behavior, and we also included a behavioral task, the BART, in our assessment of risk‐taking, whereas previous research has typically only employed parent‐, teacher‐, or self‐report questionnaires (e.g., Bimmel et al., 2008; Paysnick & Burt, 2015; Van der Graaff et al., 2016). When assessing impulsivity, a construct similar to that of risk‐taking behavior, Sharma et al. (2014) found that correlates between questionnaire measures and laboratory tasks of impulsivity are consistently low, which lead them to hypothesize that rating‐scale measures and behavioral tasks of impulsivity could measure different aspects related to daily‐life impulsive behaviors. A similar phenomenon could occur when assessing risk‐taking behavior, which could be an explanation for the differences between our findings regarding risk‐taking as an outcome, and it may raise the question whether we miss relevant elements related to daily‐life risk‐taking behavior when only using rating scales to assess risk‐taking. Future research could therefore consider combining rating scales and behavioral tasks when assessing risk‐taking, to capture a broader range of the factors resulting in daily‐life risk‐taking.

Mothers' and fathers' parenting did not significantly moderate any associations between ANS activity and risk‐taking or internalizing behavior either, when controlling the FDR, and neither did the combination of adolescent gender and mothers' or fathers' parenting. Once again, these findings contradict previous research (Cai & Tu, 2020; Dyer et al., 2016; Hinnant et al., 2015; Kassing et al., 2018), although Rudd et al. (2017) did not find a moderating effect of intrusive parenting on the association between baseline RSA and externalizing or internalizing behavior either, neither for girls nor for boys. Given that previous research has examined different types of parenting behavior, it could be the case that moderating effects only exist for specific parenting behaviors and not for others. For example, Cai and Tu (2020) suggest that parental monitoring may become particularly important during adolescence, but we did not examine that specific behavior in our current study. Again, the type of risk‐taking or internalizing behavior might also be of importance, given that Kassing et al. (2018) only found a significant moderating effect of parental inconsistent discipline when predicting reactive but not proactive aggression. Finally, some scholars have suggested that during adolescence, the influence of parents on their children may decrease while the influence of peers may increase (Bahr et al., 2005; Bauman et al., 2001), although other theoretical and empirical findings contradict this (e.g., DeVore & Ginsburg, 2005; Evans et al., 2013; Sangawi et al., 2018). Either way, investigating the role of peers on the associations between ANS activity and risk‐taking and internalizing behavior might be an important avenue for future research, given that particularly risk‐taking behaviors in adolescence are predominantly carried out in a peer‐group context (Steinberg, 2004). Taken together, we did not find evidence for a protective effect of high AUTH‐SW parenting, leading to low risk‐taking or internalizing behavior regardless of adolescents' ANS activity, nor did we find evidence for high AUTH‐S to lead to increased risk‐taking and internalizing behavior, particularly in dysregulated adolescents. Furthermore, our findings do not suggest that moderating effects of parenting, gender or a combination of both may be able to explain the inconsistent findings regarding the existence or direction of associations between heart rate or RSA and risk‐taking or internalizing behavior.

The current study's strengths include examining ANS activity in relation to general risk‐taking behavior in a large community sample, assessing risk‐taking behavior with both rating scales and a behavioral task, and assessing parenting styles of both mothers and fathers. Limitations include only having assessed resting ANS activity; it remains unknown whether our findings would have been the same in adolescents facing acute stress. Also, we examined risk‐taking and internalizing behavior variables, but we cannot know whether higher risk‐taking or internalizing behavior will later lead to more severe risk‐taking or internalizing problems. Finally, the study sample consisted disproportionately of well‐educated, (moderately) wealthy families, limiting the generalizability of our results. Particularly given that previous research has highlighted differences in associations between ANS activity and risk‐taking and internalizing behavior (Dietrich et al., 2007), future research should examine these associations in community samples that more closely represent the general population.

In conclusion, we found some evidence for an association between higher RSA and higher risk‐taking, specifically risk‐taking behavior measured with the BART and sensation seeking, but we found no evidence for associations involving heart rate or between ANS activity and internalizing behavior. We did not find any moderation effects of adolescent gender or maternal or paternal parenting style either. Taken together, these findings suggest that ANS activity may not be a particularly relevant factor for mild to moderate risk‐taking or internalizing behaviors in adolescents from the general population, regardless of their gender or the type of parenting they experience.

## CONFLICT OF INTEREST

The authors declare no conflicts of interest.

## AUTHOR CONTRIBUTIONS

**Lisa Loheide‐Niesmann:** Conceptualization; Formal analysis; Methodology; Writing‐original draft; Writing‐review & editing. **Tanja G. M. Vrijkotte:** Conceptualization; Data curation; Funding acquisition; Methodology; Resources; Supervision; Writing‐review & editing. **Susanne R. De Rooij:** Conceptualization; Supervision; Writing‐review & editing. **Reinout W. Wiers:** Conceptualization; Methodology; Resources; Writing‐review & editing. **Anja Huizink:** Conceptualization; Methodology; Supervision; Writing‐review & editing.

## Supporting information

Supplementary Material
