# Supplementary material for: Associations between autonomic nervous system activity and risk‐taking and internalizing behavior in young adolescents
Source: Psychophysiology. 2021 Jun 18;58(9):e13882. doi: 10.1111/psyp.13882 (PMC8459221; doi:10.1111/psyp.13882)
Supplement: Supplementary file 1 — Supplementary Material [file PSYP-58-e13882-s001.docx]

**Associations between Autonomic Nervous System Activity and Risk-Taking and Internalizing Behaviour in Young Adolescents**

**Supplemental materials**

| **Table 5.**  Demographic characteristics of BART completers and non-completers. | | | | | | | |
| --- | --- | --- | --- | --- | --- | --- | --- |
|  | BART completers | | |  | BART non-completers (n = 68) | | |
|  | *n* | Frequency (%) | Mean (*SD*) |  | *n* | Frequency (%) | Mean(*SD*) |
| Gender (% girls)  Age  Pubertal stage  Girls: % menstruating  Boys: % voice break | 807  805  393  402 | 49.3  5.3  2.7 | 11.78 (0.36) |  | 68  68  33  35 | 48.5  21.2  11.5 | 12.04 (0.32) |
| Ethnicity | 807 |  |  |  | 68 |  |  |
| % Dutch |  | 78.2 |  |  |  | 52.9 |  |
| % Moroccan |  | 2.1 |  |  |  | 8.8 |  |
| % Surinamese |  | 3.3 |  |  |  | 10.3 |  |
| % Turkish |  | 1.0 |  |  |  | 2.9 |  |
| % Other non-Western |  | 4.1 |  |  |  | 14.7 |  |
| % Other Western |  | 11.3 |  |  |  | 10.3 |  |

**3. *Unadjusted* Results**

The study results reported below are not yet adjusted for multiple testing. When adjusting the results below, using the Benjamini-Hochberg procedure with an accepted false discovery rate of 10% to control the false discovery rate (FDR), none of the significant associations described below remain significant. For each significant moderation effect (before controlling the FDR), the adjusted *p*-value after controlling the FDR is reported as well.

- 1. **Moderation Analyses**
     1. **Gender as a moderator in the associations between ANS activity and risk-taking and internalizing behavior.** Adolescent gender moderated the relationship between RSA and impulsivity (*R^2^* = .02; *R^2^* change = .01; F(8, 866) = 2.56, *p =* .01; unadjusted *p-*value interaction = .03). In boys, higher RSA was associated with slightly higher impulsivity, whereas in girls higher RSA was associated with lower impulsivity, although neither of the regression coefficients for RSA reached significance (Table 6). Also, this moderation effect of gender became non-significant when controlling the FDR (adjusted *p* = .31). None of the other associations between ANS activity and risk-taking or internalizing behavior were moderated by adolescent gender, neither before nor after controlling the FDR.
     2. **Parenting styles as moderating variables in the associations between ANS activity and risk-taking and internalizing behavior.** Mothers AUTH-SW significantly moderated the associations between both RSA and heart rate and anxiety sensitivity (heart rate: *R^2^* = .05; *R^2^* change = .01; F(9, 851) = 5.08, *p* < .001; unadjusted *p*-value interaction = .03; RSA: *R^2^* = .05; *R^2^* change = .01; F(9, 851) = 4.85, *p* < .001; unadjusted *p*-value interaction = .04). Simple slopes analyses revealed that in adolescents with mothers who use little AUTH-SW, lower RSA and higher heart rate were associated with higher anxiety sensitivity, whereas no significant associations between ANS activity and anxiety sensitivity were found in adolescents of mothers with mean or higher levels of AUTH-SW (Table 6). However, the moderation effect of maternal AUTH-SW became non-significant when adjusting the FDR (heart rate: unadjusted *p* = .03, adjusted *p* = .31; RSA: unadjusted *p* = .04, adjusted *p* = .18). Fathers’ AUTH-S moderated the association between heart rate and hopelessness (*R^2^* = .03; *R^2^* change = .0; F(9, 792) = 2.85, *p* < .01; unadjusted *p*-value interaction = .04). Simple slopes analyses suggested that in adolescents of highly AUTH-S fathers, higher heart rate may be associated with slightly higher hopelessness, whereas in adolescents of fathers with little AUTH-S, higher heart rate was associated with slightly lower hopelessness. However, neither of the regression coefficients for heart rate reached significance and when controlling the FDR, the moderation effect became non-significant as well (unadjusted *p* = .04; adjusted *p* = .40). None of the other associations between ANS activity and risk-taking or internalizing behavior variables were moderated by mothers’ or fathers’ parenting, neither before nor after controlling the FDR.
     3. **Interactions between adolescent gender and parenting styles as moderators in the associations between ANS activity and risk-taking and internalizing behavior.** The three-way interaction of RSA, adolescent gender, and fathers’ AUTH-S was significantly associated with sensation seeking (*R^2^* = .07; *R^2^* change = .01; F(12, 789) = 5.20, *p* < .001; unadjusted *p*-value interaction = .02), and so was the three-way interaction of heart rate, adolescent gender, and fathers’ authoritarian parenting (*R^2^* = .08; *R^2^* change = .01; F(12, 789) = 5.34, *p* < .001; unadjusted *p*-value interaction = .04). Only in boys whose fathers were high in AUTH-S were higher RSA and lower heart rate significantly associated with higher sensation seeking (RSA: *B(SE)* = 0.24 (0.05), *p* < .001; heart rate: *B(SE)* ***=*** -0.02(0.00), *p* = .001). When controlling the FDR, both three-way interactions became non-significant, however (RSA: unadjusted *p* = .02, adjusted *p* = .88; heart rate: unadjusted *p* = .04; adjusted *p* = .51).
        Furthermore, the three-way interaction of heart rate, gender, and fathers’ AUTH-S was significantly associated with anxiety sensitivity (*R^2^* = .06; *R^2^* change = .01; F(12, 789) = 3.85, *p* < .001; unadjusted *p*-value interaction = .04). Only in girls of fathers who used little AUTH-S was heart rate significantly associated with anxiety sensitivity, with higher heart rate being related to higher anxiety sensitivity (*B(SE)* = 0.01(0.00), *p* = .03). However, this three-way interaction also became non-significant when controlling the FDR (unadjusted *p* = .04, adjusted *p* = .72). None of the three-way interactions including mothers’ parenting or fathers’ AUTH-SW were associated with adolescent risk-taking or internalizing behavior, neither before nor after controlling the FDR.

| **Table 6.** Results of simple slope analyses for all significant moderation effects (separately for each predictor). | | | |
| --- | --- | --- | --- |
| Outcome | Predictor | B (*SE*) | *p** |
| Impulsivity  *R^2^* = .02  F(8, 866) = 2.56  *p =* .01 | Constant  Age  Gender  Maternal education  Paternal education  Financial status  Puberty  RSA  RSA x gender  RSA  Boys  Girls | .294 (.706)  .014 (.058)  -.123 (.069)  -.052 (.037)  -.038 (.035)  -.020 (.020)  -.001 (.011)  .085 (.060)  -.188 (.087)  .085 (.060)  -.103 (.064) | .68  .80  .08  .15  .29  .32  .94  .15  **.03**  .15  .11 |
| Anxiety sensitivity  *R^2^* = .05  F(9, 851) = 4.85  *p* < .001 | Constant  Age  Gender  Maternal education  Paternal education  Financial status  Puberty  Maternal authoritative parenting  RSA x maternal authoritative parenting  RSA  Low (+ 1 *SD*)  *M*  High (- 1 *SD*) | .807 (.663)  -.035 (.054)  .208 (.065)  -.110 (.034)  -.020 (.035)  -.033 (.019)  -.012 (.010)  .002 (.004)  .015 (.007)  -.119 (.059)  -.035 (.040)  .049 (.054) | .23  .52  **.001**  **.001**  .56  .08  .25  .62  .04  .**04**  .38  .37 |
| Anxiety sensitivity  *R^2^* = .05  F(9, 851) = 5.08  *p* < .001 | Constant  Age  Gender  Maternal education  Paternal education  Financial status  Puberty  Maternal authoritative parenting  Heart rate x maternal authoritative parenting  Heart rate  Low (+ 1 *SD*)  *M*  High (- 1 *SD*) | .735 (.664)  -.027 (.054)  .201 (.065)  -.116 (.034)  -.017 (.035)  -.031 (.019)  -.012 (.010)  .002 (.004)  -.001 (.000)  .009 (.003)  .004 (.002)  -.001 (.000) | .27  .61  **.002**  **.001**  .63  .10  .24  .61  **.03**  **.008**  .09  .76 |
| Hopelessness  *R^2^* = .03  F(9, 792) = 2.85  *p* < .01 | Constant  Age  Gender  Maternal education  Paternal education  Financial status  Puberty  Paternal authoritarian parenting  Heart rate x paternal authoritarian parenting  Heart rate  Low (+ 1 *SD*)  *M*  High (- 1 *SD*) | .169 (.420)  -.014 (.034)  .089 (.042)  -.007 (.022)  .011 (.021)  -.034 (.012)  -.004 (.007)  .010 (.005)  .001 (.000)  -.003 (.002)  .000 (.001)  .002 (.002) | .69  .67  **.03**  .77  .61  **.005**  .58  **.03**  **.04**  .13  .84  .22 |
